# Effects of Enhanced External Counterpulsation on Heart Rate Recovery in Patients with Coronary Artery Disease

**Published:** 2018-01

**Authors:** Akram Sardari, Seyed Kianoosh Hosseini, Ali Bozorgi, Masoumeh Lotfi-Tokaldany, Hakimeh Sadeghian, Mostafa Nejatian

**Affiliations:** *Tehran Heart Center, Tehran University of Medical Sciences, Tehran, Iran.*

**Keywords:** *Coronary artery disease*, *Counterpulsation*, *Angina pectoris*, *Heart rate*

## Abstract

**Background: **Enhanced external counterpulsation (EECP) reduces angina pectoris, extends time to exercise-induced ischemia, and improves quality of life in patients with symptomatic stable angina. We aimed to evaluate the effects of EECP on heart rate recovery in patients with coronary artery disease (CAD).

**Methods: **Between January 2011 and March 2013, a total of 34 consecutive patients (24 male, 70.6%) with symptomatic CAD, who were candidated for EECP, prospectively received 35 sessions of 1-hour EECP therapy per day, 6 days per week. The patients underwent echocardiography and a symptom-limited modified Bruce exercise test before and after EECP. Left ventricular ejection fraction (LVEF), resting and peak exercise heart rates, systolic blood pressure, heart rate at 1 and 2 minutes of recovery, exercise duration, workload, and first- and second-minute heart rate recovery were measured before EECP and compared with those after EECP.

**Results: **The mean age of the patients (70.6% men) was 64.82 ± 8.28 years. After EECP, exercise duration increased significantly from 6.48 ± 2.76 minutes to 9.20 ± 2.71 minutes (p value < 0.001). Maximum workload increased significantly (4.44 ± 1.28 vs. 5.65 ± 1.77 METS; p value < 0.001). The LVEF increased from 42.65 ± 11.82% to 44.26 ± 11.86% (p value < 0.001). The resting systolic blood pressure decreased significantly from 125.59 ± 22.35 mmHg to 116.26 ± 14.93 mmHg (p value = 0.013). The increase in the first- and second-minute heart rate recovery after EECP was not statistically significant.

**Conclusion**: The results of the present study showed that exercise duration, maximum workload, and the LVEF might increase significantly after EECP. The increase in the first- and second-minute heart rate recovery after EECP was not statistically significant.

## Introduction

An impaired heart rate response in standard exercise testing has been introduced as an important marker of higher total mortality and increased risk of coronary artery disease (CAD).^1^^-^^3^ Heart rate recovery after exercise is a predictor of mortality, independent of the angiographic severity of coronary disease.^4^ Also abnormal heart rate recovery in the exercise stress test predicts high-risk findings on single-photon emission computed tomography myocardial perfusion imaging in men.^5^ Decreased vagal activity results in an increased heart rate during exercise, and increased vagal activity after exercise decreases the heart rate: this is known as heart rate recovery.^6^ Although a reduction in heart rate recovery after exercise is mainly due to disturbances in the cardiac autonomic nervous system, the effects of other factors such as the severity of ischemia on this index have also been investigated.^7^^, ^^8^

It has been shown that enhanced external counterpulsation (EECP) is a noninvasive technique that prompts coronary artery collateral development, angiogenesis, training-like effect, and improved endothelial function.^9^ Previous studies have demonstrated that EECP reduces angina pectoris, extends time to exercise-induced ischemia, and improves the quality of life in patients with symptomatic stable angina. The present study was designed to evaluate the effects of EECP on heart rate recovery in patients with symptomatic CAD. 

## Methods

This prospective, single-center, cross-sectional study enrolled 34 adults with symptomatic CAD (having chest pain), who were referred for EECP therapy to Tehran Heart Center, Tehran University of Medical Sciences, between January 2011 and March 2013. The inclusion criteria were comprised of stable patients aged over 20 years, documented CAD based on prior myocardial infarction or coronary artery angiography (stenosis > 70% in at least 1 of the major cardiac epicardial arteries), New York Heart Association (NYHA) functional class II or higher, and ability to exercise continuously for more than 3 minutes. The exclusion criteria consisted of cardiac arrhythmias (frequent premature ventricular contractions, frequent premature atrial contractions, and atrial fibrillation or flutter), heart transplantation, myocardial infarction within the recent 3 months, moderate to severe aortic insufficiency, thrombophlebitis confirmed by Doppler sonography prior to EECP, uncontrolled hypertension (systolic > 180 and diastolic > 110 mmHg), permanent pacemaker or intracardiac defibrillator, pregnancy, anticoagulation therapy with an international normalized ratio greater than 3, abdominal or thoracic aortic aneurysm, and refusal to sign informed consent. This study complies with the Declaration of Helsinki, and the study protocol was approved by the Review Board of Tehran Heart Center. 

All the included patients were visited in the outpatient clinic by an expert cardiologist. Clinical evaluations; physical examinations; and 2D, color, and Doppler echocardiography were done before and after the completion of the treatment sessions. A patient had a positive family history of CAD if myocardial infarction or sudden death occurred before the age of 55 years in the father or any other male first-degree relative, or before the age of 65 years in the mother or any other female first-degree relative. A smoker was defined as a person who regularly smoked cigarettes or who had stopped smoking within the past 1 month. Patients with a history of taking antihyperlipidemic drugs, total cholesterol level of 200 mg/dL or higher, or low-density lipoprotein level equal to or greater than 130 mg/dL were defined as hypercholesterolemic. 

All the patients received 35 sessions of 1-hour EECP therapy per day, 6 days per week. EECP consisted of an air compressor, a treatment table, a control console, and 3 integrated air cuffs (MC2 model, VAMED Company, China). These compressive cuffs were wrapped around the patient’s lower extremities and buttocks. A well-trained nurse set the cuff inflation and deflation to the cardiac cycles guided by electrocardiography. Diastolic pressure augmentation during EECP was monitored using finger plethysmography. The cuffs were timed to inflate sequentially from the calf to the buttock after the onset of diastole and then to deflate simultaneously prior to the beginning of systole. The pressure applied to the cuffs was between 220 and 300 mmHg. After the completion of 35 sessions of EECP, clinical evaluations, physical examinations, and echocardiographic measurements were repeated in the last visit for each patient. 

Echocardiography was carried out for the evaluation of the left ventricular ejection fraction (LVEF) (by modified Simpson biplane method), severity of valvular disease, and sizing of the thoracic aorta. The patients underwent a symptom-limited modified Bruce exercise test.^10^ The exercise tolerance test was terminated with 1-minute cooldown in recovery. Resting heart rate and systolic blood pressure, peak exercise heart rate and systolic blood pressure, heart rate 1 and 2 minutes after finishing the exercise, workload, and exercise duration were measured. A positive exercise test was defined as an additional horizontal or downsloping ST-segment depression equal to or greater than 1 mm. Heart rate recovery was calculated as the difference in the heart rate at peak exercise and 1 minute and 2 minutes of recovery. 

The continuous variables with a normal distribution were presented as means ± SDs. To describe the variables that were not normally distributed (mainly heart rate recovery), we used medians (25th and 75th percentiles). The categorical variables were summarized by absolute frequencies and percentages. Changes in the mean of the values before and after treatment were tested using the paired *t*-test or the Wilcoxon signed-rank test as appropriate**. **We compared changes in the following variables before EECP and after the completion of 35 sessions of EECP: LVEF, resting heart rate and systolic blood pressure, peak exercise heart rate and systolic blood pressure, heart rate 1 and 2 minutes after finishing the exercise, exercise duration, and heart rate recovery. 

Because we hypothesized that the response of patients with low LVEFs to EECP might be different from that of patients with higher LVEFs, we also performed similar comparisons in subgroups of patients with LVEFs equal to or greater than 40% and LVEFs less than 40%. 

For the statistical analyses, the IBM SPSS Statistics for Windows, version 20.0, (Armonk, NY: IBM Corp.) was used. P values equal to or less than 0.05 were considered statistically significant.

## Results

From the 34 included patients, 24 (70.6%) were men. The demographic and clinical characteristics of the patients are presented in Table 1. The mean age of the patients was 64.82 ± 8.28 years. The mean LVEF was 42.65 ± 11.82%, and 11 (32.4%) patients had LVEFs less than 40 %. Among the 34 patients, a history of coronary artery bypass graft surgery was reported in 26 (76.5%) patients, 4 of whom also had a history of percutaneous coronary intervention. The remaining 8 patients were not a candidate for revascularization. 

**Table 1:** Demographic and clinical characteristics of the study patients*

Age (y)	64.82±8.28
Sex, male	24 (70.6)
Family history of CAD	11 (32.4)
Smoking	5 (14.7)
Hypertension	17 (50.0)
Dyslipidemia	20 (58.8)
Diabetes mellitus	13 (38.2)
Prior CABG	26 (76.5)
CABG + PCI	4 (11.8)
Left ventricular ejection fraction < 40%	11 (32.4)
Extent of native coronary artery disease	
Two-vessel disease	5 (14.7)
Three-vessel disease	29 (85.3)
Drugs	
Nitrate	32 (94.1)
Beta-blockers	28 (82.4)
ASA	26 (76.5)
ACE inhibitors	5 (14.7)
Statins	31 (91.2)
Diuretics	19 (55.9)
ARB	17 (50.0)
ADP inhibitors	11 (32.4)
Calcium channel blockers	8 (23.5)
Anticoagulant agents	7 (20.6)
Antihyperglycemic agents	7 (20.6)

CAD, Coronary artery disease; CABG, Coronary artery bypass graft surgery; PCI, Percutaneous coronary intervention; ASA, Acetylsalicylic acid; ACE, Angiotensin-II converting enzyme; ARB, Angiotensin II receptor blockers; ADP, Adenosine diphosphate receptor

* Data are presented as mean±SD or n (%).

After EECP, 22 (64.7%) patients showed improvement in chest pain in terms of at least a 1-grade reduction in the NYHA functional class. Prior to the EECP course, 20 (58.8%) patients were in the NYHA functional class III and 14 (41.2%) were in the NYHA functional class II. After the completion of EECP, the NYHA class decreased to I in 7 (50%) patients among those with the NYHA class II and remained II in the other 7 patients. The NYHA class also decreased from III to II in 16 (80%) patients and to class I in 1 (5%) patient with the NYHA class III prior to EECP. The mean of the NYHA class decreased significantly from 2.53 ± 0.61 before EECP to 1.85 ± 0.56 after EECP (p value < 0.001). 

The changes in the outcomes following the EECP course compared to the baseline are presented in Table 2. The mean LVEF and exercise duration increased significantly after EECP. Exercise duration increased by more than 60 seconds in 30 (88.2%) patients. The maximum workload improved significantly from 4.44 ± 1.28 METS to 5.65 ± 1.77 METS (p value < 0.001). The resting systolic blood pressure decreased significantly after EECP. The mean resting heart rates did not change following EECP. Also after EECP, heart rate recovery, measured in the first- and second-minute post exercise, did not show significant changes (Table 2). 

Among the 34 patients, 23 (67.6%) patients had LVEFs equal to or greater than 40% and 11 (32.4%) had LVEFs less than 40%. With regard to this classification, while exercise duration and maximum workload increased significantly in both groups, the resting systolic blood pressure decreased significantly only in the patients with LVEFs equal to or greater than 40% (Table 3). The mean LVEF also increased significantly in the group of patients with LVEFs of 40% or higher (from 49.89 ± 4.23% before EECP to 51.74 ± 3.49% after EECP; p value = 0.001). The change in exercise duration was not statistically significantly different between these 2 subgroups. The patients with LVEFs less than 40% showed a nonsignificant increase in heart rate recovery in the first minute post exercise after the completion of the EECP course. 

**Table 2:** Comparison of the exercise test-related variables before and after EECP*

	Pre EECP	Post EECP	P value
Ejection fraction (%)	42.65±11.82	44.26±11.86	< 0.001
Exercise duration (min)	6.48±2.76	9.20±2.71	< 0.001
Resting heart rate (bpm)	77.09±13.68	76.91±17.96	0.934
Peak exercise heart rate (bpm)	103.68±15.36	106.50±14.39	0.322
Heart rate 1 minute after exercise (bpm)	95.21±13.55	95.85±14.01	0.773
Heart rate 2 minutes after exercise (bpm)	83.26±15.11	83.79±16.35	0.818
Maximum workload (METS)	4.44±1.28	5.65±1.77	< 0.001
Heart rate recovery (bpm)			
First minute	6.5 (0.75-16)	8.5 (5.75-18.25)	0.109
Second minute	20 (9.75-30.5)	23 (13-33.5)	0.400
Resting systolic blood pressure (mmHg)	125.59±22.32	116.26±14.93	0.013
Maximum systolic blood pressure (mmHg)	134.56±19.75	133.53±19.09	0.734

EECP, Enhanced external counterpulsation

* Data are presented as mean±SD or median (25th and 75th percentiles).

**Table 3:** Comparison of the exercise test-related variables before and after EECP with regard to the pre-EECP ejection fraction

	EF < 40% (n=11)			EF ≥ 40% (n=23)		
	Pre EECP	Post EECP	P value	Pre EECP	Post EECP	P value
Ejection fraction (%)	27.50 ±6.98	28.64±6.36	0.059	49.89±4.23	51.74±3.49	0.001
Exercise duration (min)	5.41±2.44	8.43±2.84	0.001	6.99±2.81	9.58±2.63	< 0.001
Resting heart rate (bpm)	83.36±16.82	82.36±19.40	0.573	74.09±11.09	74.30±17.06	0.944
Peak exercise heart rate (bpm)	107.18±12.98	108.82±13.93	0.702	102.00±16.37	105.32±14.78	0.370
Heart rate 1 minute after exercise (bpm)	101.73±14.64	99.27±17.17	0.555	92.09±12.10	94.22±12.33	0.435
Heart rate 2 minutes after exercise (bpm)	91.00±14.68	89.91±22.18	0.800	79.57±14.16	80.87±12.25	0.641
Maximum workload (METS)	4.05±1.09	5.53±1.63	0.005	4.63±1.34	5.71±1.86	0.003
Heart rate recovery (bpm)						
First minute	4 (1-8)	7 (6-16)	0.066	10 (0-17)	9 (5-21)	0.455
Second minute	17 (11-23)	18 (10-25)	0.445	21 (9-33)	28 (14-35)	0.626
Resting systolic blood pressure (mmHg)	120.45±14.05	112.73±14.38	0.243	128.04±25.26	117.96±15.20	0.032
Maximum systolic blood pressure (mmHg)	127.73±15.71	122.73±16.49	0.452	137.83±20.93	138.70±18.35	0.791

*Data are presented as means±SD medians (25th and 75th percentiles).

EECP, Enhanced external counterpulsation

## Discussion

The results of the present study showed that exercise duration and maximum workload increased significantly after EECP irrespective of the LVEF. The first- and second-minute heart rate recovery increased nonsignificantly after EECP. 

To our knowledge, none of the previous studies has focused mainly on the evaluation of the effects of EECP on heart rate recovery. Indeed, the previous studies have evaluated the effects of EECP on exercise duration, time to ST depression, and workload.^11^^-^^14^ We observed that heart rate recovery, 1 minute after exercise, increased from a median of 6.5 bpm to 8.5 bpm after EECP. Although the level of this improvement was not significant, probably due to the small sample size, this finding illustrates the positive effect of EECP on exercise heart rate response in patients with CAD. This effect was more prominent in the patients with LVEFs less than 40%. Our results also demonstrated a significant rise in maximum workload after EECP compared to the baseline in our CAD patients.

Previous studies have demonstrated that EECP increases exercise duration and improves functional status in congestive heart failure patients aged at least 65 years.^11^^, ^^12^^, ^^15^ Abbottsmith et al.^10^ reported that the patients in their EECP group had a progressive increase in exercise duration over time, while the figure decreased progressively in the control subjects. Chiming in with these findings, in the present study, exercise duration increased by more than 60 seconds in over 88.2% of the patients and this effect was observed in those with either LVEFs less than 40% or LVEFs of 40% or higher.

In the MUST-EECP trial,^13^ the first randomized controlled study to evaluate the effects of EECP, over 70% of the study patients with symptomatic CAD had undergone prior cardiopulmonary bypass surgery or angioplasty and the results showed that the use of EECP in this population reduced angina and extended the time to ischemia in the exercise tolerance test. Similar to that study, more than 75% of our study subjects also had previously undergone coronary artery bypass graft surgery and we found that it increased exercise duration. Micheals et al.^16^ studied the effects of EECP on myocardial perfusion in patients with stable angina. Compatible with the results of our study, the authors reported that exercise duration increased, while the average heart rate recovery remained unchanged after EECP.

One of the limitations of the present study is its small sample size, which may have affected the results of the study. Increased heart rate recovery 1 and 2 minutes after exercise did not constitute statistical significance in this study, which may have been due to our small sample size. Another drawback of note is related to the heterogeneity of the patients with CAD enrolled in this study, which precluded us from presenting conclusions in a specific group of patients with CAD. Although lack of long-term follow-up is not a limitation to this study, it could have revealed the effects of EECP on the quality of life and stability of the positive effects of EECP in the long term. 

## Conclusion

The present study evaluated the effects of enhanced external counterpulsation on exercise capacity and heart rate recovery in patients with symptomatic coronary artery disease. The results revealed that enhanced external counterpulsation may increase exercise duration and maximum workload significantly. Moreover, in the patients with left ventricular ejection fractions less than 40%, heart rate recovery in the first minute post exercise showed an increase toward the significant level when compared to the rate before enhanced external counterpulsation.

## Notes:

***This paper should be cited as: ***Sardari A, Hosseini SK, Bozorgi A, Lotfi-Tokaldany M, Sadeghian H, Nejatian M. Effects of Enhanced External Counterpulsation on Heart Rate Recovery in Patients with Coronary Artery Disease. J Teh Univ Heart Ctr 2018;13(1):13-17.
